# Facile Synthesis of Polyethylene Glycol@Tannin-Amine Microsphere towards Cr(VI) Removal

**DOI:** 10.3390/polym13071035

**Published:** 2021-03-26

**Authors:** Chengbing Yuan, Yan Zhang, Jinshui Yao, Qinze Liu, Fan-Gong Kong

**Affiliations:** State Key Laboratory of Biobased Material and Green Papermaking, School of Materials Science and Engineering, Qilu University of Technology (Shandong Academy of Sciences), Jinan 250353, China; sdnyycb@126.com (C.Y.); 17864181164@163.com (Y.Z.); yaojsh@qlu.edu.cn (J.Y.)

**Keywords:** tannin-amine composite, polyethylene glycol, Cr(VI) removal

## Abstract

Herein, a synthetic strategy for a rough microsphere Cr(VI)-adsorbent via the reaction of tannic acid (TA) and 1,6-hexanediamine (HA) and using polyethylene glycol (PEG) as surface modifier was presented. This adsorbent was characterized by a Fourier Transform Infrared spectrometer (FTIR), thermogravimetic analysis (TGA), X-ray photoelectron spectroscopy (XPS), etc. Certain factors, including contact time, PEG@poly(tannin-1,6-hexanediamine) (PEG@PTHA) dosage, initial concentration, and experimental temperature affecting the Cr(VI) adsorption performance of adsorbent were explored. PEG@PTHA can adsorb Cr and the Cr(VI) was reduced up to Cr(III) due to the existence of phenolic hydroxyl groups. Its adsorption capacity can reach up to 300 mg/g within 10 min and approximately 100% removal percentage below the initial concentration of 100 mg/L. Its behavior matched well with the Langmuir isotherm model and pseudo-second-order kinetic model. A PEG@PTHA adsorbent with maximum adsorption capacity (450 mg/g) has great prospects in Cr(VI)-sewage treatment.

## 1. Introduction

Cr(VI) poses an enormous threat to human health due to its high carcinogenicity; Cr(VI) was produced in the industrial production of leather tanning, textile dyeing. In view of its high toxicity and refractory degradability in solution, it poses a huge threat to human health [1,2,3]. Cr generally exists in two valence states: trivalent and hexavalent [4]. Cr(III) is an essential element for human growth. In order to meet the emission standard (0.05–0.1 mg/L), many methods including membrane separation, advanced oxidation, biological treatment, and ozone treatment were used to remove or reduce Cr(VI) [5]. Based on the limitations of the above-mentioned methods (high cost, low adsorption efficiency, and poor operability), more suitable routes need to be explored urgently.

Comparing with photocatalytic degradation [6], oxidation-reduction, and coprecipitation [5,7,8], adsorption is regarded as a promising water treatment method in view of its low energy consumption and easy operation [9,10]. The development of cheap and efficient adsorbent has become the top priority in the industry, which is directly related to its practical application. So far, various novel adsorbents have been reported, for example, inorganic adsorbents including activated carbon and graphene have some disadvantages of low removal capacity and poor selectivity [11,12]. As for the organic adsorbent including fluorescent chitosan-based hydrogel and epichlorohydrin crosslinked polyethylenimine-chitin adsorbent, the complexity of the preparation process limits their wide application [13,14]. Hence facile preparation and low cost are considered as key factors for the design of Cr(VI)-adsorbent.

Tannic acid (TA) is a natural polyphenolic compound widely found in various plants [15]. The catechol groups present in TA react with primary amine groups to form a new type of macromolecular structure polymer, and it is rich in phenolic hydroxyl (ph-OH) and –NH_2_ groups, which provides a new idea for the design of new adsorbents [16,17]. Liu et al. prepared a polytannin-1,6-hexamethylenediamine adsorbent with an adsorption capacity of 200 mg/g to remove Cr(VI) [18]. In addition, polyethylene glycol (PEG) has the advantage of easy solubility and has great potential for removing Cr(VI) [19].

In this work, a tannin-amine microsphere was fabricated via an environmentally friendly and straightforward preparation method with the help of polyethylene glycol (PEG) [17]. PEG, a potential Cr(VI)-adsorbent, can be mixed in a tannin-amine system to form rough porous microspheres and increase the rate and amount of adsorption [20]. Various characterization methods were selected to evaluate structural performance and adsorption capacity, and adsorption experiments were conducted sequentially. Besides, the adsorption model including kinetics, isotherms, and thermodynamics were further studied in the following.

## 2. Experimental Section

### 2.1. Materials

Poly(ethylene glycol) (PEG, AR) and Potassium dichromate (K_2_Cr_2_O_7_, AR) were purchased from Maclean, Tannic acid (TA, AR) and 1,6-hexanediamine (HA, AR) were supported by Aladdin. The ultrapure water (UW 18.25 MΩ cm) was utilized in the following experiments.

### 2.2. General Characterization

Thermogravimetric analysis (TGA, TGA-1, Mettler Toledo, Columbus, OH, USA) was used to investigate the difference in the thermal stability of temperature range of 45–800 °C (10 °C/min). A Fourier transform infrared spectrometer (FT-IR, Thermo Nicolet, Waltham, MA, USA) was used to investigate the characteristic chemical bonds of materials. Microtopography and element content were examined with a scanning electron microscope (SEM, Quanta 200, Hillsboro, OR, USA) and an energy dispersive spectrometer (EDS, Quanta 200, Hillsboro, OR, USA). X-ray photoelectron spectroscopy (XPS, Thermon Scientific, Waltham, MA, USA) was used to investigate the change of elements. An ultraviolet spectrophotometer (UV, UV-2550, Tokyo, Japan) was used to investigate the actual concentration of Cr(VI) solution.

### 2.3. Preparation of PEG@PTHA

**Figure 1:**
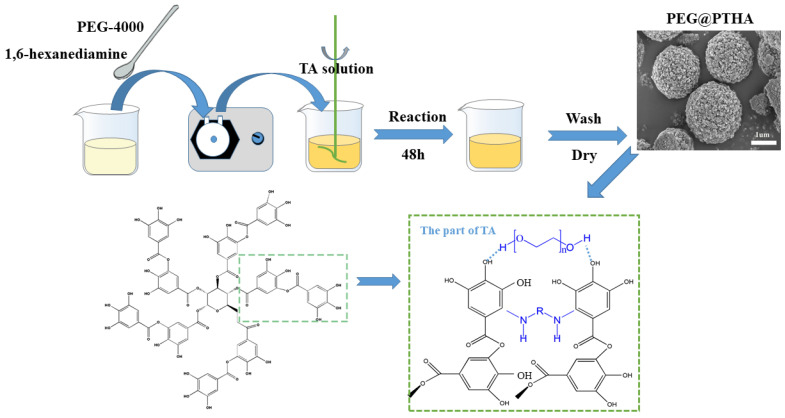
Schematic diagram of preparation procedure and possible preparation mechanism of the PEG@PTHA composite adsorbent.

By adjusting the PEG/TA weight ratio and fixing the HA/TA mole ratio (12.5:1), four microsphere adsorbents were fabricated at room temperature including 0:1 (0-PEG@PTHA), 0.5:1 (PEG@PTHA), 1:1 (1-PEG@PTHA), and 2:1 (2-PEG@PTHA). Taking PEG@PTHA as an example, (1) 0.5 g PEG and 0.85 g HA were added into 50 mL UW and then ultra-sounded to disperse evenly; (2) 1.0 g TA was charged into 50 mL UW and dispersed uniformly; (3) the mixture of PEG and HA was added into the above TA solution by drop. The sediment was separated and alternately washed with UW and ethanol absolute three times. The resultant PEG@PTHA composite was finally dried via freeze-drying for next use. The preparation process of PEG@PTHA and its possible reaction mechanism can be seen in Figure 1.

### 2.4. Adsorption Experiment

All concentrations of the solution used were diluted from a pre-prepared high concentration solution (2000 mg/L), and the pH value was regulated by HCl (1 mol/L) and NaOH (1 mol/L). Experiments were executed to explore the effects of significant parameter concluding contact time (0–24 h), pH value (2–7), initial concentration (60–160 mg/L), and temperature. For this purpose, 100 mL of Cr(VI) solution was taken in a 200 mL grinding mouth bottle with a certain amount of adsorbent. The bottles were put into a shaker for 24 h or 48 h. The concentration of Cr(VI) solution was measured by UV instrument according to the 1,5-diphenylcarbazide method.

The adsorption behavior of the adsorbent was evaluated with the following Equations (1) and (2):

(1) $$q_{t}=(C_{0}-C_{t})V/m$$

where *q_t_* (mg/g) is the adsorption capacity at contact time *t* (s); *q_e_* (mg/g) can be obtained at equilibrium time; *C*_0_ (mg/L) and *C_t_* (mg/L) are the initial and real-time concentrations, respectively; *V* (L) is the simulated wastewater volume; and *m* (g) represents the weight of PEG@PTHA.

(2) $$R\%=(C_{0}-C_{e})/C_{0}\times 100\%$$

where *R* (%) is the removal rate; and *C*_0_ (mg/L) and *C_e_* (mg/L) are the initial and equilibrium concentrations of Cr(VI), respectively.

## 3. Results and Discussion

### 3.1. SEM-EDS Characteristic

**Figure 2:**
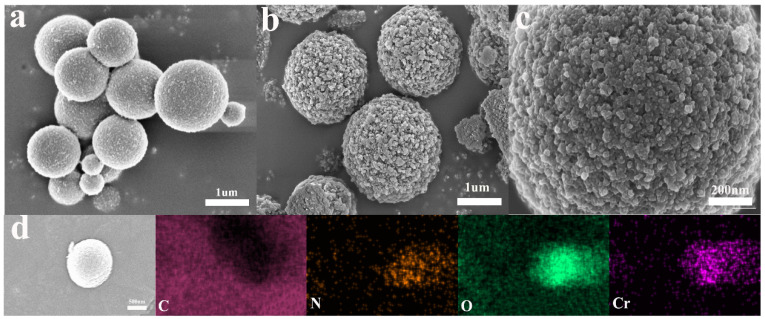
(**a**) SEM image of PTHA, (**b**) SEM image PEG@PTHA, (**c**) partial enlarged SEM image of PEG@PTHA, (**d**) EDS image of used PEG@PTHA.

The difference between 0-PEG@PTHA and PEG@PTHA in morphology is shown in Figure 2a,b. The surface of PEG@PTHA is much rougher than that of 0-PEG@PTHA, which can be due to the presence of PEG. Its high dissolubility and leaving of the PEG-PTHA system cause the existence of this rough surface (Figure 2c). The EDX image of PEG@PTHA after loading Cr(VI) is presented in Figure 2d. In addition to C, N, and O, there were Cr elements loaded on the adsorbent surface. It can be concluded that Cr distributed evenly over the surface of the adsorbent. 

### 3.2. FT-IR Characteristic

**Figure 3:**
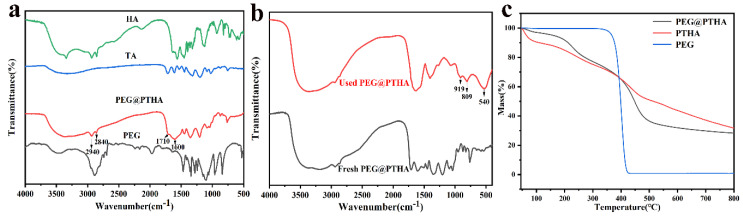
(**a**) FTIR spectrogram of HA, TA PEG, and PEG@PTHA, (**b**) FT-IR spectrogram of Fresh PEG@PTHA and used PEG@PTHA, (**c**) TGA curve of PEG@PTHA, PTHA, and PEG.

The FT−IR spectrum of PTHA and PEG@PTHA can be seen in Figure 3a. The peak at 1080–1150 cm^−1^, which was attributed to stretching vibration of C−O−C in PEG, can be found at 1035–1140 cm^−1^ in PEG@PTHA, the peaks at 2840 and 2940 cm^−1^ were attributed to the stretching vibration of C-H in HA, there were two small adsorption peaks in the same range in PEG@PTHA. The wide characteristic peak at 3550–3200 cm^−1^ resulted from the stretching vibration of O−H in TA and –NH in HA. The stretching vibration of C=O and the benzene ring in PEG@PTHA were located at 1710 and 1600 cm^−1^, respectively. PEG was attached successfully to the surface of PTHA microspheres and they combined well with each other. As can be seen in Figure 3b, the peak at 540 cm^−1^ was attributed to the vibration of Cr=O, and the peaks at 919 and 809 cm^−1^ were attributed to the phenolic hydroxyl group that interacts with Cr [21].

### 3.3. TGA Analysis

The thermal performances of PTHA and PEG@PTHA were investigated by using heat constantly from 45−800 °C in the N_2_ atmosphere (10 k/min). The downtrend of PTHA and PEG@PTHA before 150 °C was attributed to the weight loss of the combined water. The thermostability of PEG@PTHA was better than PTHA before 400 °C, however, due to the molecular chain of PEG being broken at 400 °C, the heat resistance of PTHA was better after 400 °C, which also confirmed the presence of PEG (Figure 3c).

### 3.4. Effect of Contact Time

**Figure 4:**
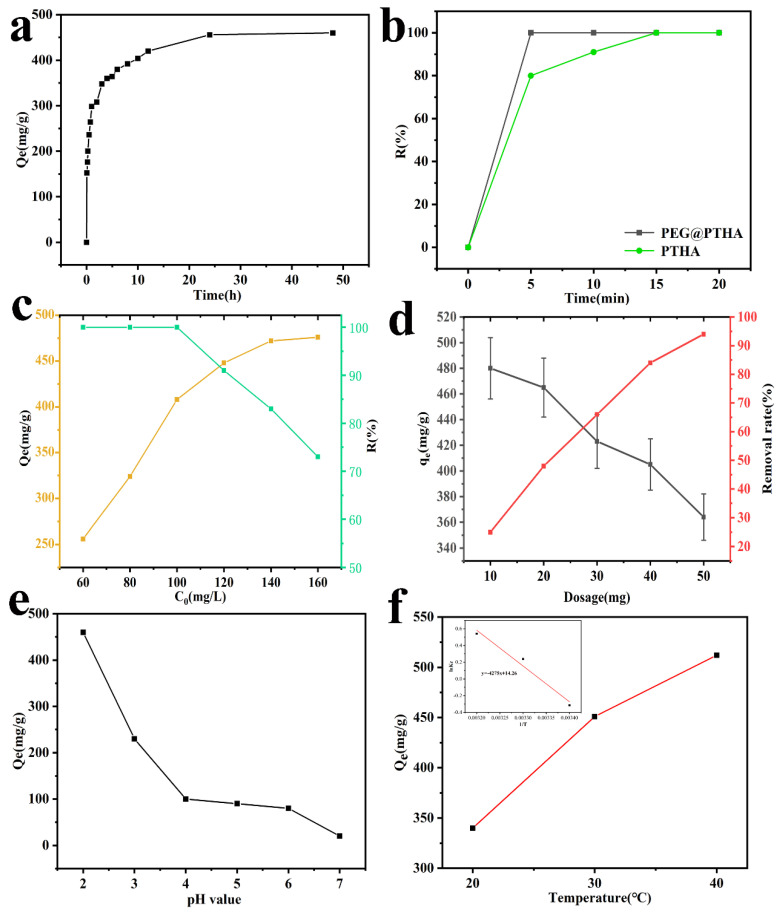
(**a**) effects of contact time (0–48 h, *C*_0_ = 200 mg/L, T = 303 K, dos = 25 mg, V = 100 mL, pH = 2), (**b**) the difference of removal rate between PTHA and PEG@PTHA (*C*_0_ = 60 mg/L, pH = 2, T = 303 K, dos = 25 mg, V = 100 mL), (**c**) effect of initial concentration (*C*_0_ = 60–160 mg/L, T = 303 K, dos = 25 mg, t = 24 h, V = 100 mL), (**d**) effect of different adsorbent dosage (10–50 mg, *C*_0_ = 200 mg/L, T = 303 K, V = 100 mL, pH = 2), (**e**) effect of PH value (*C*_0_ = 200 mg/L, T = 303 K, dos = 25 mg, V = 100 mL, t = 24 h), (**f**) effect of different environmental temperature (*C*_0_ = 200 mg/L, dos = 25 mg, V = 100 mL, t = 24 h).

As can be seen in Figure 4a, the adsorption capacity increases with the extension of contact time, the adsorption capacity can reach up to 200 mg/g after 10 min. On account of the decrease of the adsorption active site, the removal rate of Q_t_ slowed down, the equilibrium was basically reached at 24 h. In view of this situation, we selected 24 h as the contact time in the subsequent adsorption experiments. The fast adsorption capacity of PEG@PTHA is shown in Figure 4b. PEG@PTHA achieved an almost 100% removal percentage much sooner compared to the pure PTHA adsorbent under the same experimental conditions (100 mL, 100 mg/L, 30 °C). The reason for this phenomenon is due to the existence of the rough surface of PEG@PTHA, which can increase the chance of contact between the active site and chromium ions, thus possessing a faster removal property.

### 3.5. Effect of Initial Concentration

The comparison of different Cr(VI) concentrations (60–160 mg/L) is presented in Figure 4c. It can be concluded that the adsorption capacity of PEG@PTHA enhanced with the increase of ion concentration. While the initial concentration was less than 100 mg/L, the removal percentage of Cr(VI) can reach up to nearly 100%, which is owed to the strong interaction between amino and carboxyl groups in PEG@PTHA and Cr(VI). The decrease in the removal percentage and the increase in the adsorption amount can be discovered as the initial concentration condensed.

### 3.6. Effect of Different Adsorbent Dosage

As shown in Figure 4d, the effect of different PEG@PTHA dosages was studied, it revealed the relationship between adsorbent dosage and removal amount. With the increase of PEG@PTHA dosage, the removal amount decreased from 480 mg/g to 360 mg/for a constant concentration of the Cr(VI) solution. When the amount of PEG@PTHA microspheres increases, the removal amount (or percentage) increases in the meanwhile. Yet, the adsorption capacity per unit mass of PEG@PTHA decreased and the utilization of PEG@PTHA became greatly lower.

### 3.7. Effect of pH Value

On account of the influence of pH value on the surface functional group state of PEG@PTHA, it was significant for Cr-removal to control the pH of the aqueous solution. The adsorption capacity decreases gradually with the enhancement of pH value, in the aqueous solution of pH = 2–6, Cr ions existed primarily in the form of HCrO_4_^−^. The capacity to adsorb HCrO_4_^−^can be enhanced by the protonation of –OH and –NH_2_ groups to –OH_2_^+^and –NH_3_^+^. With the increase of pH, the degree of protonation goes down, resulting in the attenuation of adsorption capacity.

### 3.8. Adsorption Isotherm Analysis

The adsorption isotherm model is a significant technology that can be used to speculate the adsorption behavior of the adsorption isotherm model is appropriate for single-layer adsorption that occurs on a completely uniform surface. The equation is as follows:

Langmuir model

(3) $$C_{e}/Q_{e}=1/(K_{L}Q_{m})+C_{e}/Q_{e}$$

in which *Q_m_* (mg/g), *Q_e_* (mg/g), and *C_e_* (mg/L) represent maximum adsorption capacity, actual capacity, and residual concentration of Cr(VI), respectively. *K_L_* (L/mg) is the adsorption equilibrium factor for site affinity.

Freundlich’s adsorption model assumes that the adsorbate reacts on the nonuniform surface by multi-layer adsorption, which indicates that the combined site is inequitable and co-dependent. The equation is as follows:

Freundlich model

(4) $$\log Q_{e}=\log K_{F}+1/n\log C_{e}$$

in which *K_F_* ((mg/g)/(mg/L)) and 1/*n* are the Freundlich factors of adsorption capacity and adsorption strength, the numerical value of *n* shows the advantage of adsorption. The Langmuir and Freundlich models were used to fit the experimental data. Judging from the correlation coefficient, the R^2^ in the Langmuir adsorption isotherm model can better fit the experimental data, and the calculated *Q_m_* is more consistent with the experimental data in Table 1.

### 3.9. Adsorption Kinetic Model

So as to explore the kinetic mechanism of the adsorption process, the conventional kinetic model is used to analyze experimental data, Lagergren’s pseudo-first-order and pseudo-second-order models can be used to describe this adsorption process.

Lagergren pseudo-first-order

(5) $$\log(q_{e}-q_{t})=\log q_{e}-k_{1}t/2.303$$

Lagergren pseudo-second-order

(6) $$t/q_{t}=1/(k_{2}q_{e}{}^{2})+t/q_{e}$$

Therein, *k*_1_ (1/min) and *k*_2_ (g/mg min) respectively correspond to the adsorption rate constant of Lagergren’s pseudo-first-order and Lagergren pseudo-second-order. The *Q_m_* and *Q_t_* correspond to the adsorption capacity of equilibrium and any sampling time, respectively. The rate constant is applied to analyze the adsorption mechanism.

The values of *Q_e_*, *k*_1_, *k*_2_, and R^2^ are listed in Table 2, the Lagergren pseudo-second-order possesses higher R_2_, which can better describe the kinetics of Cr(VI) removal by PEG@PTHA.

### 3.10. Intra-Particle Diffusion Model

**Figure 5:**
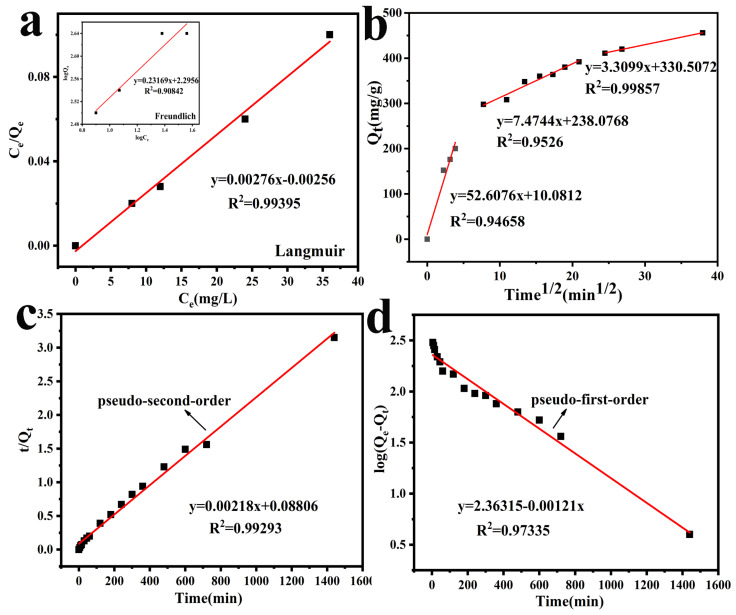
(**a**) Langmuir isotherm model and Freundlich isotherm model, (**b**) The intra-particle diffusion model, (**c**) Pseudo-second-order model, (**d**) Pseudo-first-order model.

We explained the importance of diffusion toward adsorption through an intra-particle diffusion model. The Weber–Morris equation is as follows:

(7) $$q_{t}=K_{id}t^{1/2}+C$$

where *K_id_* represents the intra-particle diffusion speed constant, and *C* represents the boundary diffusion effect. The linear fitting equation of the diffusion in the particle can be obtained via the linear fitting of *t*^1/2^ and *q_t_*. The fitting result can be seen in Figure 5b. It displayed three discrete lines, which suggested that the adsorption process was operated by various procedures. *K_id_* was particularly large in the first section and revealed a sharp interaction in the second section. The line fitting began to level off. This phenomenon is different from the developmental trend of the first stage The decrease in *K_id_* indicates the drop in the severity of adsorption. The gentle phenomenon of the third section indicates the ending of adsorption. When the concentration of the target solution reduced and the diffusion rate slowed down, an equilibrium was attained at the end.

### 3.11. Thermodynamic Study

Temperature will affect the adsorption rate and physical structure of the PEG@PTHA on the target pollutant. On the basis of the change in temperature, the adsorption thermodynamics can provide information about the thermal properties and spontaneity of the removal process of Cr(VI) in the simulated sewage.

The relationship equation of the thermodynamic constants Δ*G* (Gibbs free energy), Δ*H* (standard enthalpy change), and Δ*S* (standard entropy change) is as follows:

(8) ∆G=∆H−T·∆S

(9) $$K_{c}=C_{a}/C_{e}=(C_{0}-C_{e})/C_{e}$$

where *C_a_* (mg/L) represents the amount of Cr(VI) adsorbed on the PEG@PTHA, and *C_e_* (mg/L) represents the concentration of the residual CR in solution.

The following is the Van Hoff equation:

(10) $$\ln K_{c}=-\Delta H/RT+\Delta SR$$

where Δ*G* can be determined by using Equation (7) according to the fitting of a linear equation to calculate the Δ*H* and Δ*S* concrete numerical value (Figure 4e). As can be seen in Table 3, the value of Δ*G* was negative, which demonstrated the feasibility and spontaneity of the adsorption process, the value of Δ*H* was positive, which indicated that the adsorption process was endothermic, and Δ*S* > 0 revealed that the degree of chaos in the adsorption process increased.

### 3.12. Effect of Competing Ions

**Figure 6:**
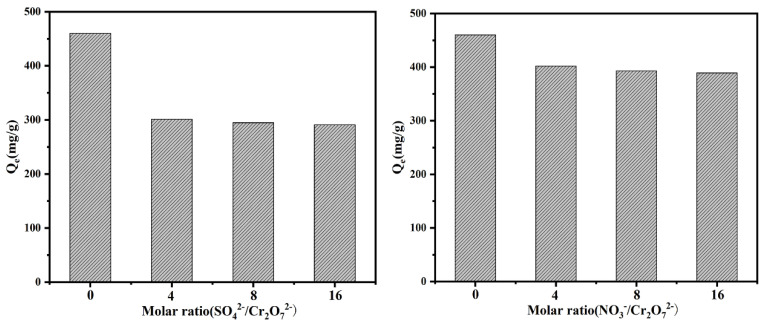
The effect of competing ions on the removal behavior towards Cr(VI).

The presence of co-existing ions can affect the adsorption capacity obviously, as can be seen in Figure 6. The addition of NO_3_^−^ and SO_4_^2−^ decreased the value of *Q_e_*, in other words, the adsorption capacity decreased with the interference of both of them. Further increasing the proportion of investment, the value of *Q_e_* changed less. Comparing with the weak electrostatic force between the active site and HCrO_4_^−^, SO_4_^2−^ had a clear competitive advantage, comparatively, the addition of NO_3_^−^ did not have an excessive effect due to its weak competition.

### 3.13. XPS Analysis and Conjectural Adsorption Mechanism

**Figure 7:**
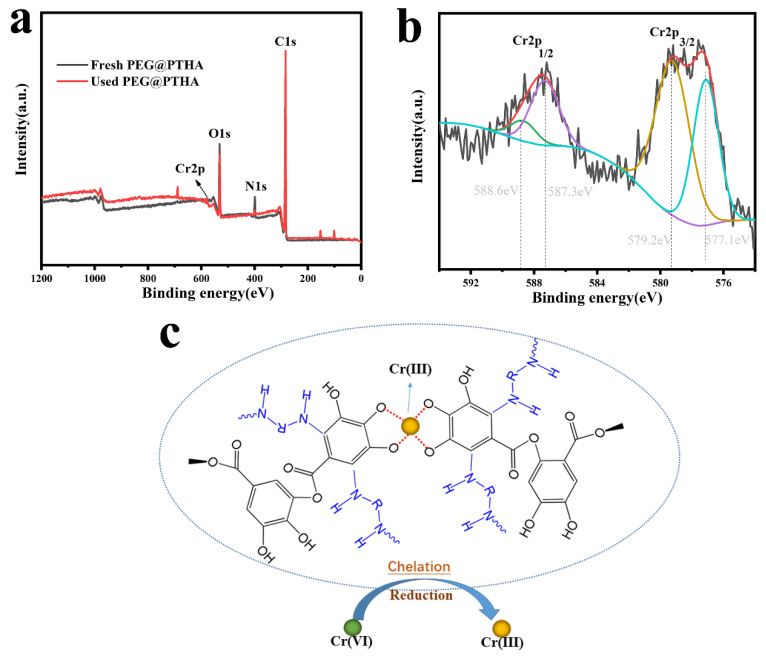
(**a**) the XPS analysis of fresh PEG@PTHA and used PEG@PTHA, (**b**) XPS Cr2p spectrogram on the used PEG@PTHA, (**c**) conjectural mechanism of adsorption.

As is shown in Figure 7, the removal mechanism of Cr was investigated by XPS analysis, the appearance of a Cr2p peak on the used PEG@PTHA revealed that the Cr was successfully captured. According to the Cr2p enlarged spectrogram, the peaks at 588.6 eV and 577.1 eV were attributed to Cr(III), while the peaks located in 587.3 eV and 579.2 eV were assigned to Cr(VI). The most of toxic Cr(VI) was reduced to the weakly toxic Cr(III) due to the existence of the –OH group, and the Cr(III) was chelated on the PEG@PTHA during the adsorption-reduction process. Comparing with the other kinds of adsorbents in Table 4, the PEG@PTHA in this work possesses a higher adsorption amount and a brighter future for wastewater treatment.

## 4. Conclusions

In this work, a PEG@PTHA adsorbent microsphere with a rough surface was fabricated successfully and used as an adsorbent for Cr(VI)-wastewater treatment. Comparing with other kinds of adsorbents(Table 4), it possessed an approximate 99% removal rate at a low initial concentration (100 mg/L) and maximum adsorption capacity (450 mg/g), moreover, its adsorption capacity could reach up to 300 mg/g within 10 min. The adsorption process better fitted with the pseudo-second-order model and Langmuir isotherm. There was not only physical adsorption but also chemical adsorption, virulent Cr(VI) was reduced to the less harmful Cr(III) during the adsorption process, and PEG@PTHA appears to have a high-efficiency Cr(VI) competitive adsorption against anions (NO_3_^−^ and SO_4_^2−^). It can be concluded that the PEG@PTHA adsorbent with advantages of facile preparation, high selectivity, and low cost possesses excellent prospects in Cr(VI)-contained wastewater treatment.

## Figures and Tables

**Table 1:** The parameters of isotherm models for removal of Cr by PEG@PTHA.

Langmuir Isotherm Model				Freundlich Isotherm Model		
**Adsorbate**	***K_L_* (L/mg)**	***q_m_* (mg/g)**	**R^2^**	***K_F_* (mg/g)/(mg/L)^1/n^**	**1/n**	**R^2^**
Cr(VI)	1.08	362	0.99393	197	0.23169	0.90842

**Table 2:** The parameters of the kinetic models for removal of Cr by PEG@PTHA.

Pseudo-First-Order				Pseudo-Second-Order		
**Adsorbate**	***K*_1_ (h^−1^)**	***q_e_* (mg/g)**	**R^2^**	***K*_2_ (m/mg h^−1^)**	***q_e_* (mg/g)**	**R^2^**
Cr(VI)	0.00279	229	0.97335	0.0000544	458	0.99293

**Table 3:** Thermodynamic Parameters of Cr(VI) Adsorption on PEG@PTHA microsphere.

Temperature (K)	Δ*G* (KJ/mol)	Δ*H* (KJ/mol)	Δ*S* (J/molK)
293	−1.36		
303	−2.56	35.5	1.68
313	−4.17		

**Table 4:** Comparison of adsorption capacity with other adsorbents from previous studies.

Adsorbent	Adsorption Capacity	Reference
lignin–graphene oxide composite nanospheres	154 mg/g	[22]
two-dimensional (2D) Ti3C2Tx MXene nanosheets	104 mg/g	[23]
functionalized tannin-chitosan bentonite composite	197 mg/g	[24]
Nitrogen-Doped Carboxylated Porous Carbon	104 mg/g	[25]

## Data Availability

Data sharing is not applicable.
